# Hepatoid Adenocarcinoma of the Stomach: A Challenging Diagnostic and Therapeutic Disease through a Case Report and Review of the Literature

**DOI:** 10.3389/fmed.2017.00164

**Published:** 2017-09-28

**Authors:** Najla Fakhruddin, Hisham F. Bahmad, Tarek Aridi, Yara Yammine, Rami Mahfouz, Fouad Boulos, Ahmad Awada, Fadi Farhat

**Affiliations:** ^1^Department of Pathology and Laboratory Medicine, American University of Beirut Medical Center, Beirut, Lebanon; ^2^Department of Pathology, Hammoud Hospital University Medical Center, Saida, Lebanon; ^3^Faculty of Medicine, Department of Anatomy, Cell Biology and Physiological Sciences, American University of Beirut, Beirut, Lebanon; ^4^Faculty of Medicine, American University of Beirut, Beirut, Lebanon; ^5^Medical Oncology Clinic, Jules Bordet Institute, Université Libre de Bruxelles, Brussels, Belgium; ^6^Faculty of Medical Science, Department of Hematology-Oncology, Saint Joseph University, Beirut, Lebanon; ^7^Department of Hematology-Oncology, Hammoud Hospital University Medical Center, Saida, Lebanon

**Keywords:** hepatoid adenocarcinoma, stomach, alpha fetoprotein, liver metastasis, case report

## Abstract

Hepatoid adenocarcinoma of the stomach (HAS) is a rare aggressive tumor with hepatocellular differentiation. HAS often produces alpha fetoprotein (AFP) and metastasizes to the lymph nodes and the liver. Molecular studies revealed *Her2* amplification and overexpression, association with *p53* mutations, but no association with *KRAS* mutations. *EGFR* and *BRAF* mutations have not yet been evaluated in hepatoid carcinoma of the stomach so far. Hereby, we present a case of a 41-year-old female patient with HAS with high AFP level and liver metastases. Molecular analysis revealed *Her2* overexpression by immunohistochemistry (IHC), but no *EGFR, KRAS*, or *BRAF* mutations were detected. The patient underwent chemotherapy type DCX (docetaxel, cisplatinum, and capecitabine) every 3 weeks with partial response after two cycles, maintained for eight cycles, and then was on maintenance therapy with trastuzumab for 7 months before relapsing and dying 18 months from the day of diagnosis. Conclusively, HAS may be misdiagnosed as hepatocellular carcinoma; therefore, it should be considered in the differential diagnosis of multiple hepatic nodules with high AFP and no history of hepatitis, liver fibrosis or cirrhosis.

## Introduction

**Figure 1:**
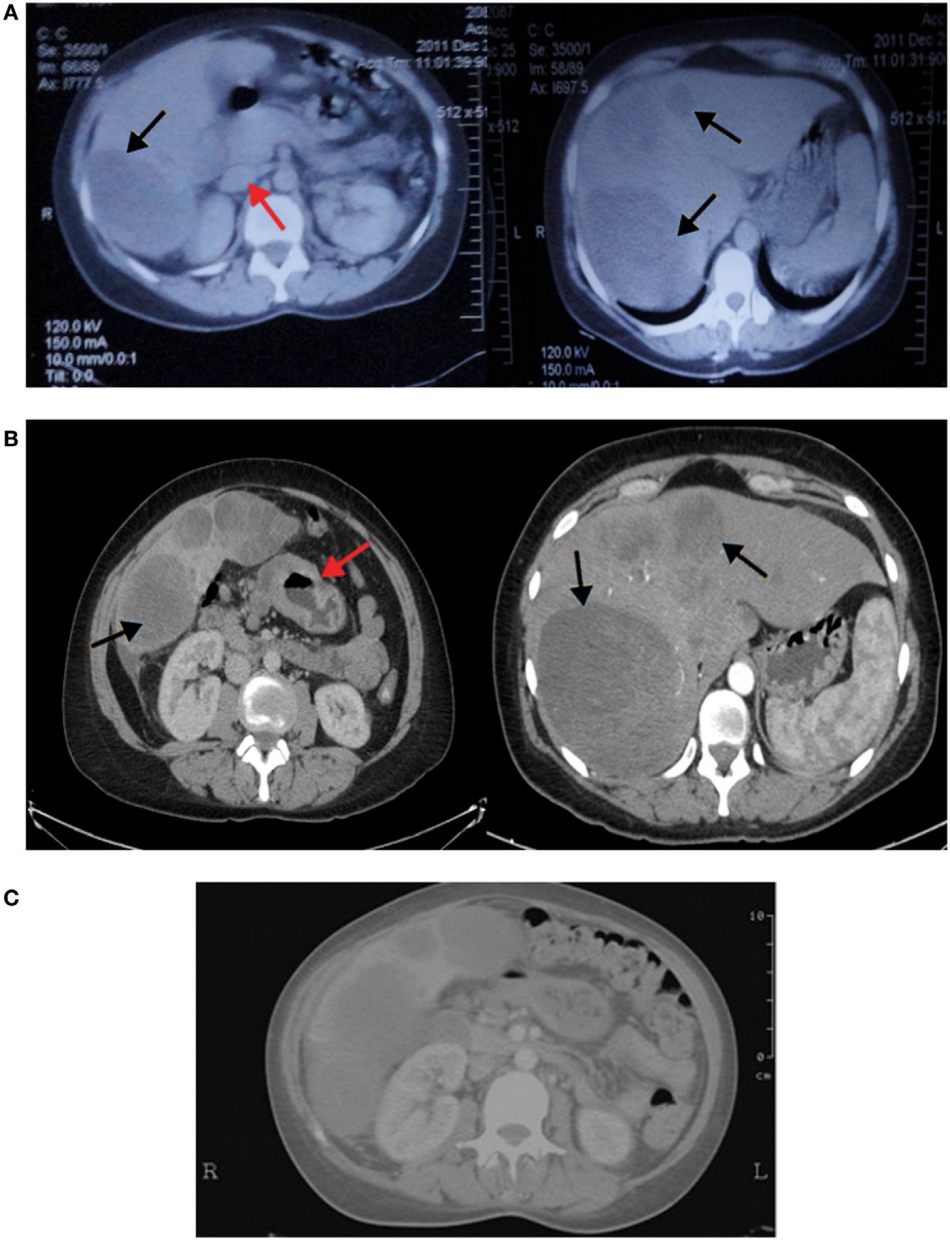
**(A)** Computed tomography (CT) of the abdomen showing the right hepatic lobe occupied by a large hypodense mass and left lobe contains a smaller scattered solid nodule (black arrows). In addition, enlarged lymph nodes at porta hepatica are seen (red arrow). **(B)** Vertical computed tomography showing heterogenously enhancing hepatic masses (black arrows) with circumferential infiltrative gastric wall thickening (red arrow). **(C)** CT scan of the abdomen showing no changes in liver metastasis with significant clearing of gastric wall thickening.

**Figure 2:**
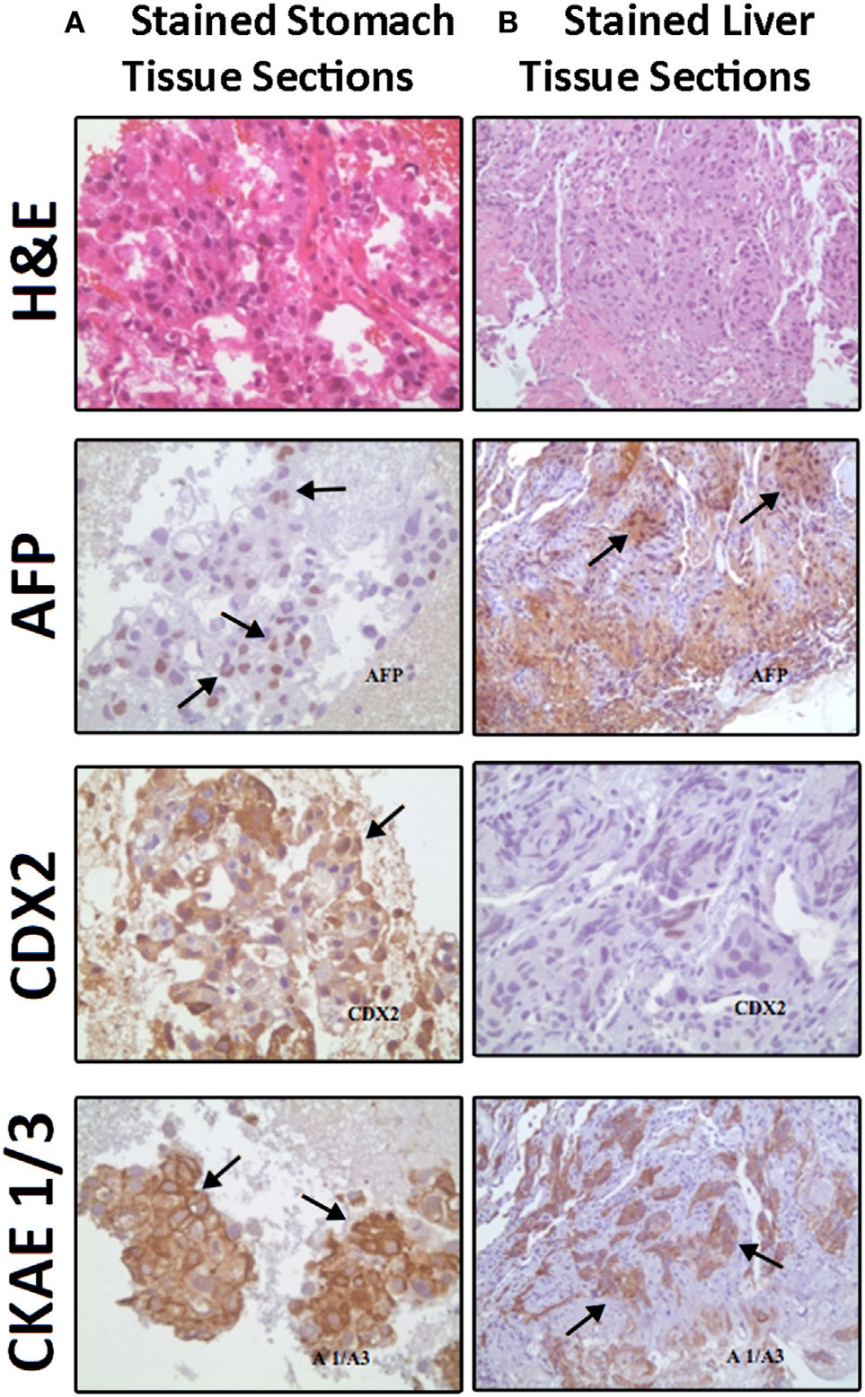
Comparable Hematoxylin and Eosin (H&E) staining and immunostaining for alpha fetoprotein (AFP), CDX2, and cytokeratin AE1/AE3 (CK AE1/3) expressions (brown color refers to positively stained cells) between **(A)** stomach biopsy tumor tissue sections (consisting mainly of hepatoid cells) on the left side and **(B)** liver fine needle aspirate tumor tissue sections (showing infiltrate of neoplastic hepatoid cells) on the right side.

A 41-year-old female, previously healthy with unremarkable past medical or surgical history and a positive maternal family history of colon cancer, presented to another hospital 4 months prior to the diagnosis of hepatoid adenocarcinoma of the stomach (HAS) with bloating and abdominal pain localized in the epigastrium and right upper quadrant, and radiating to the back especially after meals. Routine laboratory blood tests (complete blood count, electrolytes, blood urea nitrogen, and creatinine blood level) were normal. Abdominal ultrasound revealed three liver nodules in the right lobe of mixed echogenicity with the largest measuring 9.5 cm. Serological tests revealed absence of hepatitis B or C antigens. Alpha fetoprotein (AFP) level was markedly elevated (61,360 IU/L). Abdominal computed tomography (CT) scan showed a large hypodense mass in the right hepatic lobe and a smaller scattered solid nodule in the left lobe. In addition, enlarged lymph nodes at the porta hepatica were seen (Figure 1A). Fine needle aspirate of the liver masses was performed and revealed pathological features in favor of hepatocellular carcinoma diagnosis (Figure 2B).

The patient was referred to our center as having a hepatocellular carcinoma for further management and workup. At presentation, patient was hemodynamically stable with blood pressure of 120/80 mmHg, pulse rate of 84 beats per minute, respiratory rate of 16 breaths per minute, body temperature of 37.5ºC and O_2_ saturation of 99%. On physical examination, abdomen was soft with localized tenderness at the right upper quadrant, and hepatomegaly (4 cm below right costal margin) with palpable nodular consistency. Normal bowel sounds were heard on auscultation. No pulsating masses were noted in the abdomen. Otherwise, the rest of physical examination was normal with no fever or chills.

A total body vertical computed tomography was done (Figure 1B) and showed tiny subcentimetric cervical lymph nodes, minimal bilateral pleural thickening, atelectatic band of left lung base, and hepatomegaly with multiple heterogenous masses of the right and left lobes the largest measuring 12 cm. In addition, it revealed wall thickening of the stomach at the greater curvature and the antrum extending to 1.7 cm in thickness and stranding adjacent fat with perigastric, mesenteric, and hilar lymph nodes. Minimal pelvic free fluid was noted as well (Figure 1B). The serum level of Ca19-9 was markedly elevated (7,024 IU/mL), but not carcinoembryonic antigen (CEA) (2.26 IU/L) and β-HCG (1.03 IU/L). Gastroscopy was performed and showed the presence of a large ulcerated lesion with hard margins in the antrum.

A liver biopsy done outside our institution (Figure 2B) and a gastric biopsy at our institution (Figure 2A) were examined and underwent IHC markers evaluation all supporting the diagnosis of HAS (Table 1). The histology demonstrated predominance of the hepatoid features in both the liver and the gastric tissues, but no glandular formations seen (Table 1). Chronic active gastritis with *Helicobacter pylori* and intestinal metaplasia were present. *HER2/neu* amplification evaluation by IHC showed an overexpression with a score of 3+. Paraffin tissue ribbons were then obtained to perform molecular analysis for *EGFR, KRAS*, and *BRAF* on the gastric biopsy but all were wild type.

**Table 1:** Imunohistochemistry profile of the stomach and liver lesions.

IHC marker	Liver	Stomach
Alpha fetoprotein	+	+
Hep Par 1	Focal+	−
CK AE 1/3	+	+
CDX2	−	weak+, focal
CK7	−	−
CK20	−	−
Synaptophysin	−	−
Vimentin	−	−
PLAP	−	−
Carcinoembryonic antigen	Focal+	NA
HER2/neu	NA	Overexpressed

*+, positive, −, negative, NA, not available*.

The patient was started on docetaxel, cisplatin, and capecitabine (DCX). After two cycles of chemotherapy, AFP dropped to 12,100 (previously 61,360) IU/L, and CA19-9 to 6,019 (previously 7,024) IU/L. The performance status according to Eastern Cooperative Oncology Group performance scale improved from 3 before the first cycle of chemotherapy to 1 before the third. A follow up CT was performed and showed partial response of gastric wall thickening and the liver metastasis with less prominent enhancement suggestive of central necrosis (Figure 1C). After eight cycles of chemotherapy, 6 months from diagnosis, we started her on three weekly Trastuzumab as maintenance therapy in view of *HER2* amplification for 7 months. Meanwhile, follow-up CT scans showed partial response after 2, 4, and 6 months in addition to decrease in tumor markers’ (AFP and Ca19.9) levels: AFP dropped to 59.9 while CA19.9 dropped to 136.

After 7 months of Trastuzumab monotherapy and 1 year after starting chemotherapy, CT scan showed a progression of disease, with increased AFP and Ca19.9 levels. Therefore, treatment was restarted on DCX with Trastuzumab for 4 cycles. After 3 months of DCX and Trastuzumab, the disease progressed. The therapy was switched to Oxaliplatin; 5-fluorouracil; leucovorin; and trastuzumab of which she received a total of three cycles. Evaluation CT scan showed a progressive disease.

The next plan was to start the patient on Trastuzumab and CPT-11, which was shown to have promising results in therapy especially in combinations (1, 2). She received one dose of each but has deceased 2 months later from complications with an 18-month survival from the day of diagnosis.

## Background

Hepatoid adenocarcinoma of the stomach is a rare aggressive tumor with hepatocellular differentiation that often produces AFP. It occurs usually in elderly people with unclear pathogenesis. This entity was coined by Ishikura et al. in 1985 (3), and several reports and reviews were published thereafter (4, 5). HAS frequently metastasizes to the liver and the lymph nodes, and usually present as liver nodules primarily. HAS may be misdiagnosed as hepatocellular carcinoma as in the case presented here; therefore, it should be considered in the differential diagnosis of multiple hepatic nodules with high AFP, and no history of hepatitis, liver fibrosis, or cirrhosis (6, 7).

Hepatoid adenocarcinoma of the stomach exhibits a mixed tubular and/or papillary, and a hepatoid cellular pattern. *HER2* gene amplification and overexpression of its encoded protein was detected in HAS as well (8). *EGFR, KRAS*, and *BRAF* mutations are involved in the tumorigenesis of gastrointestinal carcinomas that can potentially respond to the available tyrosine kinase inhibitors (9). *EGFR* and *BRAF* have not yet been investigated in HAS tumors so far.

**Figure 3:**
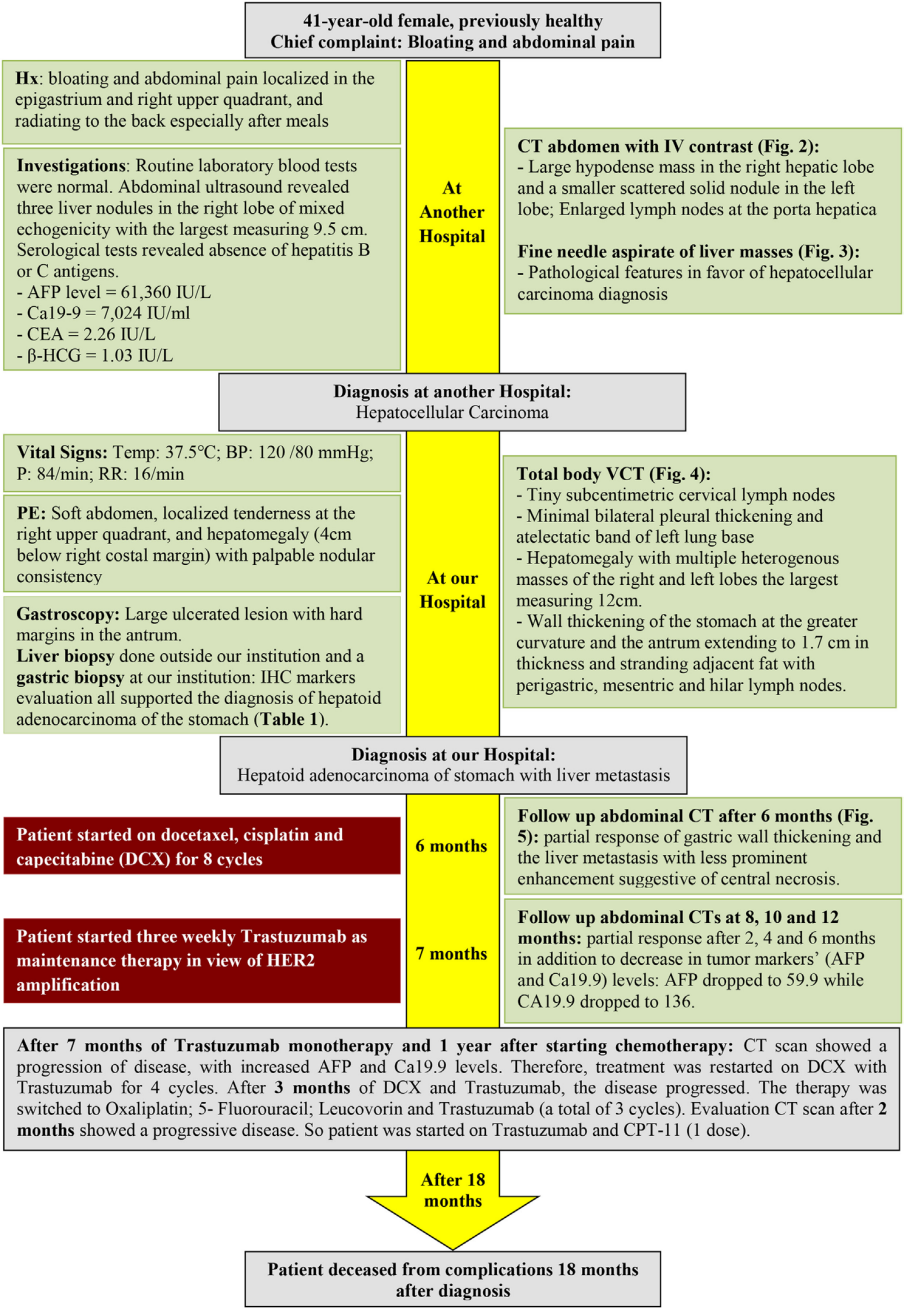
Timeline organizing main events of the case.

Multiple cases of HASs have been reported in literature; however, our paper is the first to report the youngest case of HAS with liver metastasis in a 41-year-old lady. The case was diagnosed using a panel of immunohistochemical markers and evaluated for *EGFR, KRAS*, and *BRAF* mutations, besides *HER2* overexpression by IHC. Bright field double *in situ* hybridization for *Her2* test revealed a ratio of 3.1, thus indicating a detected mutation in the *HER2/neu* gene. In addition, a comprehensive review of the literature on HAS is reported. This case report (Figure 3) was conducted and reported in accordance with CAse REports (CARE) guidelines for reporting case reports.

## Discussion

Hepatoid adenocarcinoma of the stomach are a subtype of gastric tumors with hepatoid features and frequently producing AFP proteins. Their pathogenesis and molecular biology is thought to be due to cellular trans-differentiation from glandular to hepatoid type, but this is not clear so far (3). Moreover, the role of *H. pylori* infection in this subtype of gastric carcinoma is not yet established of tumors. The HAS case presented here is the youngest in the literature so far. Some of gastrointestinal and hepatocellular carcinomas exhibit mutations in *EGFR, KRAS*, and *BRAF* genes. In case of positivity of these mutations targeted tyrosine kinase inhibitors or monoclonal antibodies could potentially be used (9). Since HAS is chemo-resistant and metastasize and recur quickly, we considered checking for the presence of any of these mutations mentioned above.

Clinically, patients’ age range from 44 to 87 years and the male to female ratio is 2.3 to 1 (5). The main symptoms are epigastric pain and general fatigue due to anemia. The majority of cases present in an advanced stage, with elevated serum AFP ranging from less than 1.0 to 700,000 ng/mL. Frequently, metastasis occurs to the lymph nodes and to the liver; one case with brain metastasis (10) and another with spleen metastasis (11) were reported.

Patient’s survival may not exceed 2 years, and the longest reported was 99 months (12). As compared to gastric adenocarcinoma with no hepatoid features regardless of AFP production, the prognosis is poorer with a 5-year survival rate of 9% (13). However, early detection followed by curative surgery can result in cure (14).

Radiographically, CT scans show eccentric gastric wall thickening. HAS appears isodense at unenhanced CT or moderately enhanced. Presence of heterogeneity correlates with the presence of hemorrhage and necrosis. The tumor size usually ranges from 1.6 to 14.0 cm, with presence of venous invasion around the primary gastric tumor or the metastatic hepatic mass (15).

Morphologically, these tumors have hepatoid cellular images that may be associated with adenocarcinoma features. Immunohistochemically, evaluation for hepatoid adenocarcinoma showed reactivity to AFP, alpha antitrypsin, alpha chemotrypsin, and CEA. In addition, albumin mRNA was detected by *in situ* hybridization indicating their hepatocellular differentiation (4). Molecular studies revealed that HAS originates from the endodermal stem cells that can have differentiation into hepatoid and/or intestinal cell lines (3). *HER2* amplification and protein overexpression was found in intestinal type gastric cancer and was reported in HAS recently. EGFR signaling is known to drive *via* the MAPK pathway where *KRAS* and *BRAF* molecules act along its downstream. Mutations in their genes are detected in gastrointestinal tumors (9). Investigation revealed no *EGFR, KRAS*, or *BRAF* mutations, but knowing that these three mutation tend to be expressed mutually exclusive as in the case of lung adenocarcinoma and thyroid papillary cancer we tested this case but all were wild type. However, this is not sufficient to negate their occurrence. Considering the therapeutic potential of TKI’s for these targets and the aggressiveness of this tumor, further investigation in a larger group is worth considering.

Treatment consists mainly of radical surgery when feasible followed by chemotherapy, including cisplatin, epirubicin, 5-fluorouracil, and leucovorin or a combination of platinum with fluoropyrimidine and then Paclitaxel (14). Median overall survival and progression-free survival after treatment are 8.03 (95% CI: 6.59–9.47) and 3.47 months (95% CI: 0.65–6.29), respectively (14). Our patient has crossed a period of 18 months survival and a period of partial response during 8 months.

## Concluding Remarks

The unique and rareness entity of HAS makes the diagnosis of this type of tumors a dilemma for the pathologist and the clinician and may lead to a misdiagnosis between HCC and HAS. The treatment of metastatic disease remains to be defined. The diagnosis of HAS should be considered in case of multiple hepatic tumors with elevated AFP and the performance of an upper gastrointestinal endoscopy should be considered to rule out a gastric HAS. Whether targeted therapy is efficacious in the setting of HAS is not clear so far. The need of further studying the molecular pathogenesis and the efficacy of chemotherapy and targeted therapy in HAS is of utmost importance for a better approach and management.

## Consent for Publication

Written informed consent was obtained from the patient for publication of this case report and accompanying images. A copy of the written consent is available upon request for review by the Editor-in-Chief of this journal.

## Ethics Statement

This study was carried out in accordance with the recommendations of the Institutional Review Boards (IRB) of the American University of Beirut Medical Center (AUBMC) and Hammoud Hospital University Medical Center (HHUMC) with written informed consent from the included subject. The patient gave written informed consent in accordance with the Declaration of Helsinki. The protocol was approved by the Institutional Review Board of AUBMC and HHUMC. Written informed consent was obtained from the patient for participation in this case report study. A copy of the written consent is available upon request for review by the Editor-in-Chief of this journal.

## Author Contributions

FF and NF worked on the case study conception and design and contributed to the data collection, pathological slides review, selection of tissue for the molecular analysis, and data analysis. TA and YY were responsible for getting the clinical data from medical records of the hospital and writing section “Case Presentation.” NF and RM worked on the pathological slides review, selection of tissue for the molecular analysis, and data analysis. NF worked also on the histology figures and performed the molecular analysis experiments and molecular data analyses. RM, FB, and AA provided other authors with explanations about the case reported. HB worked on the figures illustrations and case study timeline presentation. FF, NF, and HB were responsible for writing the discussion and editing the whole manuscript, in addition to proofreading. FF and NF were responsible for the study supervision and conduction of the whole project. All authors contributed to the drafting of the manuscript and critically revised and edited the manuscript prior to approving the final draft. All authors approved the final draft of the manuscript.

## Conflict of Interest Statement

The authors declare that the research was conducted in the absence of any commercial or financial relationships that could be construed as a potential conflict of interest.

## Abbreviations

HAS, hepatoid adenocarcinoma of the stomach; AFP, alpha fetoprotein; IHC, immunohistochemistry; BDISH, bright field double *in situ* hybridization; CEA, carcinoembryonic antigen; CT, computed tomography; VCT, vertical computed tomography; DCX, docetaxel, cisplatin and capecitabine; ECOG, Eastern Cooperative Oncology Group; IRB, Institutional Review Board; AUBMC, American University of Beirut Medical Center; HHUMC, Hammoud Hospital University Medical Center.
